# Assembly of platforms for signal transduction in the new era: dimerization, helical filament assembly, and beyond

**DOI:** 10.1038/s12276-020-0391-3

**Published:** 2020-03-05

**Authors:** Hyun Ji Ha, Hye Lin Chun, Hyun Ho Park

**Affiliations:** College of Pharmacy, Chung-Ang University, Seoul, 06974 Republic of Korea

**Keywords:** Protein-protein interaction networks, Innate immunity

## Abstract

Supramolecular organizing center (SMOC)-mediated signal transduction is an emerging concept in the field of signal transduction that is ushering in a new era. The formation of location-specific, higher-order SMOCs is particularly important for cell death and innate immune signaling processes. Several protein interaction domains, including the death domain (DD) superfamily and the CIDE domain, are representative mediators of SMOC assembly in cell death and innate immune signaling pathways. DD superfamily- and CIDE domain-containing proteins form SMOCs that activate various caspases and provide signaling scaffold platforms. These assemblies can lead to signal transduction and amplification during signaling events. In this review, we summarize recent findings on the molecular basis of DD superfamily- and CIDE domain-mediated SMOC formation.

## Nonspecific immunity: Super signaling complexes

Improved understanding of large molecular signaling complexes that form during innate (nonspecific) immune responses could help develop treatments for multiple diseases including cancer. Correct cell signaling requires precise protein interactions and binding, which are mediated by specific sites on the surface of the protein molecules involved. Innate immune responses and cell death mechanisms rely on such protein interactions, and defects can cause signaling abnormalities and trigger disease. Hyun Ho Park and co-workers at Chung-Ang University in Seoul, South Korea, reviewed recent insights into the presence of supramolecular organizing centers (SMOCs), localized complexes of signaling proteins that form during immune responses. The researchers highlight existing understanding of SMOC assembly processes. A better understanding of SMOCs will help to explain enzyme activation, signal amplification and cell signaling control mechanisms.

## Introduction

Specific protein interactions are critical for proper cellular signaling, and failed and mismatched protein interactions cause signaling defects and alter the fate of cells. Protein interaction is usually mediated by protein interaction domains, a critical part of proteins involved in the binding of specific sequences to other proteins^1,2^.

Cell death and innate immune signaling pathways are important defense mechanisms against various pathogens. These processes are mediated by various complicated protein–protein interactions that transfer signals and control cellular signaling events. Many proteins participating in these cellular signaling events contain small protein interaction domains, such as the death domain (DD), death effector domain (DED), caspase-recruiting domain (CARD), PYrin domain (PYD), baculovirus IAP repeat (BIR) domain, Bcl-2 homology (BH) domain, and/or cell death-inducing DFF45-like effector (CIDE) domain. These proteins interact with specific binding partners^3–5^. Among the protein interaction domains, DD, DED, CARD, and PYD belong to the death domain (DD) superfamily. This is one of the largest protein interaction domain families, sharing sequence homology and a unifying structural feature: a six-helix bundle fold^3,6–8^.

Apoptotic DNA fragmentation is a hallmark of apoptosis and is primarily mediated by the CIDE domain-containing proteins DFF40 and DFF45 ^9^. DFF40 is an endonuclease that digests chromosomal DNA and produces nucleosomal fragments, whereas DFF45 is an inhibitor of DFF40 ^10^. Both DFF40 and DFF45 contain a CIDE domain that can mediate the interaction between two proteins, resulting in the inhibition of DFF40 nuclease activity by DFF45 ^11,12^. In addition to DFF40/DFF45, the CIDE-A, CIDE-B, and CIDE-3 proteins have been identified as having CIDE domains^11,13^. Although functional studies have shown that these three CIDE domain-containing proteins are also involved in apoptosis regulation, recent studies have indicated their role in energy metabolism, specifically their involvement in controlling the size of lipid droplets^14–16^.

Over recent decades, the interprotein interactions in the cell death and innate immune signaling pathways mediated by DD superfamily-containing and CIDE domain-containing proteins have been intensively studied. This interest is based on their functional importance in biological systems and their links to many human diseases, including cancer, obesity, and various immune diseases^7,17–22^. Studies have revealed that various signaling molecules in cell death and innate immune signaling form higher-order signaling complexes called supramolecular organizing centers (SMOCs) via DD superfamily or CIDE domains^23,24^. In addition to SMOC formation, DD superfamily-containing proteins can be assembled into various oligomerization structures. In this review, we summarize the binding strategies of the DD superfamily and CIDE domains detected thus far. We also discuss the biological significance of these assemblies during cell death and innate immune signaling events.

### Structure and function of the DD superfamily

In the early 1990s, an intracellular DD comprising ~90 amino acids was first identified and named during a cellular study on tumor necrosis factor receptor and Fas^25–27^. Since then, genetic, functional, and structural analyses have revealed similar DD-like domains in various proteins, designated DEDs^28^, CARDs^29^, and PYDs^30,31^. The subfamily classification is mainly determined by sequence homology^6^. In humans, 37 DD-containing proteins, 7 DED-containing proteins, 33 CARD-containing proteins, and 22 PYD-containing proteins have been identified thus far and found to be particularly functional during cell death and innate immunity events^4,6^. DD superfamily-containing proteins specifically interact through their DDs with other downstream DD superfamily-containing proteins, thus transferring signals through cellular signaling. In addition, DD superfamily-mediated SMOC formation is critical for activating various caspases and kinases, which are necessary for cell death and innate immunity processes^32,33^.

**Fig. 1:**
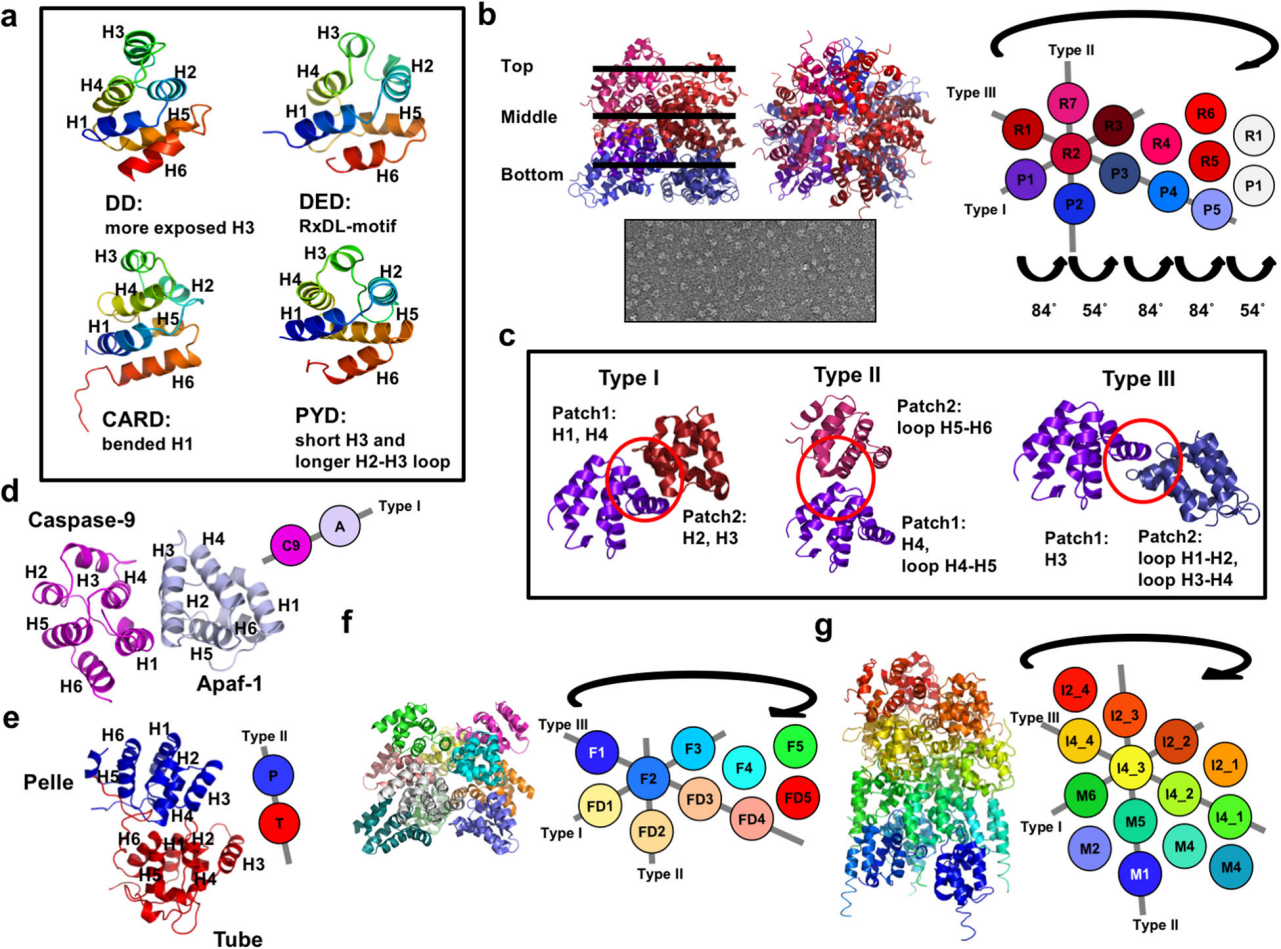
Structure of the death domain (DD) superfamily and its prototype assembly scheme. **a** The structural features of DD superfamily members. Ribbon diagrams for each subfamily in the DD superfamily are shown. Representative structures are provided showing the Fas death domain as the DD, the FADD death effector domain (DED) as the DED, the RAIDD caspase recruitment domain (CARD) as the CARD, and the NLRP3 pyrin domain (PYD) as the PYD. The distinct structural features of each superfamily are indicated. The structures in the N-terminus to the C-terminus are colored blue and red, respectively. Secondary structures, H1−H6, are indicated on the corresponding structure. **b** The first helical structure discovered in the DD superfamily. The helical assembly is formed by a heteromeric complex of RAIDD DD and PIDD DD. Top and side views of the helical structure are shown in the upper left panel. Electron microscopy image of the negatively stained RAIDD DD/PIDD DD complex is presented in the lower left panel. The bottom layer formed by five PIDD DDs, the middle layer formed by five RAIDD DDs, and the top layer formed by two RAIDD DDs are indicated in the side view of the complex structure. A schematic planar diagram showing the strategy for the helical assembly by successive screw rotations with up and down interactions in a hypothetical PR subcomplex is shown in the right panel; P and R indicate PIDD DD and RAIDD DD, respectively. **c** The three different interaction prototypes, types I, II, and III, formed by DD superfamily members. The interaction interface is shown with a red circle. The helixes and loops involved in the interaction are indicated. **d** Prototype of the heterodimeric structure of the DD superfamily formed by caspase-9 CARD and Apaf-1 CARD. Secondary structures, H1−H6, are indicated on the corresponding structure. This heterodimer is formed by type I interactions. **e** Prototype of the homodimeric structure of the DD superfamily formed by Pelle DD and Tube DD. Secondary structures, H1−H6, are indicated on the corresponding structure. This heterodimer is formed by type II interactions. **f**, **g** Other cases of helical assembly by DD superfamily members. **f** Representative helical structures formed by heteromeric Fas DD and FADD DD and **g** by heteromeric IRAK2 DD, IRAK4 DD, and MyD88 DD are shown. Schematic planar diagrams are presented to show binding strategy information; F and FD P indicate Fas DD and FADD DD, respectively; I2, I4, and M indicate IRAK2 DD, IRAK4 DD, and MyD88 DD, respectively.

The six-helix bundle fold is the common feature of the DD superfamily (Fig. 1a). The structure of the Fas DD, with the six-helix bundle fold, was the first structure among the DD superfamily-containing proteins to be identified^25^. Then, the structures of the Fas-associated DD protein (FADD) with a DED^28^; RIP-associated protein with DD (RAIDD) with a CARD^34^; and NACHT, leucine-rich repeat and PYD-containing 1 (NLRP1) with a PYD^35^ were elucidated (Fig. 1a). Although they possess a common structural fold, each subfamily has unique structural features, including a flexible and exposed third helix (H3) in DDs, an RxDL motif in DEDs, a bent first helix (H1) in CARDs, and a relatively small H3 and long H2-H3 connecting loop in PYDs (Fig. 1a).

### Prototype interactions of the DD superfamily

Each member of the DD superfamily interacts with members of its own subfamily^3,8,36–38^. The general strategy of DD superfamily assembly was revealed via a structural study of the PIDDosome core complex, a molecular complex that activates caspase-2 and is composed of RAIDD DD and PIDD DD^7^. This structural study showed that seven RAIDD DD and five PIDD DD molecules formed a circular, three-layer structure: two layers are formed by RAIDD DD and one is formed by PIDD DD (Fig. 1b). The main two layers, the bottom layer and middle layer, are formed by five PIDD DDs and five RAIDD DDs, respectively, whereas the top layer is formed by two additional RAIDD DDs (Fig. 1b). This circular structure is formed by the unique rotation and translation strategy of DD molecules. A planar schematic showed that the circular complex is constructed by five successive screw rotations of the DD molecules in the same layer around the central vertical axis. One screw rotation rotates approximately 84° and translates down the axis, and the other rotates approximately 54° and translates up the axis. These segments can form a circular structure with three 84° and two 54° rotations ((84 × 3) + (54 × 2) = 360°) (Fig. 1b). This unique circular structure formed by DDs participates in three types of interactions, namely, types I, II, and III, which are now considered the prototype interactions of the DD superfamily (Fig. 1b). In type I interactions, residues in the H1 and H4 helices of one DD interact with residues in the H2 and H3 helices of the bound DD (Fig. 1c). In type II interactions, an interface is formed by residues in the H4 helix and the H4−H5 loop of one DD and the residues in the H5−H6 loop and H6 helix of the bound DD (Fig. 1c). In type III interactions, residues in the H3 in one DD and the residues in the connecting loops, from H1−H2 and H3–H4, of the bound DD. This interaction strategy formed by the hetero DD complex was also detected in members of other subfamilies of the DD superfamily. The representative heterodimeric CARD structure of the Apaf-1 CARD and caspase-9 CARD complex revealed that the interaction is mediated by the mutual recognition of the concave surface formed by H1 and H4 in the caspase-9 CARD and the convex surface formed by H2 and H3 in the Apaf-1 CARD. These interactions indicate that this heterodimeric CARD structure is constructed via typical type I interactions (Fig. 1d)^39^. Type I-mediated homodimer CARD assembly was also discovered via a structural study of ARC CARD^40^. The homodimer interface of ARC CARD is created by salt bridges and hydrogen bonds formed between the residues in H1 and H4 of one ARC CARD and the residues in H2 and H3 of its counterpart. The type II-mediated dimeric DD structure was discovered by a structural study of Pelle DD and Tube DD (Fig. 1e)^41^. In this case, H4 and the H4−H5 loop in Tube DD interact with the H5−H6 loop in Pelle DD. Further structural study of the complex formed by DDs revealed that a similar assembly strategy, based on prototype type I, type II, and type III interactions, occurs during Fas DD/FADD DD complex formation (Fig. 1f)^17,42^, RIP1DD/FADD DD complex formation^42^, and MyD88 DD/IRAK4 DD/IRAK2 DD complex formation (Fig. 1g)^20^. This suggests that the type I-, type II-, and type III-mediated assembly strategies are common among DD superfamily members. However, atypical dimeric structures in members of the DD superfamily were also found in recent structural studies^43–45^.

### SMOC formation by the DD superfamily

The large molecular complexes formed by the helical assembly of DD superfamily members were difficult to determine by their crystal structure because it is impossible to crystallize a helical structure unless the helical periodicity happens to be an integer. With the development of advanced cryo-electron microscopy (EM) technology, many helical filament structures of the DD superfamily, important for SMOC formation, have been determined^18,19,21,46–50^. These structural studies revealed that members of the DD superfamily use a common assembly mechanism, detected during the structural study of the RAIDD DD/PIDD DD complex, to form the helical filament structure in SMOCs. This mechanism is formed via type I, II, and III interactions.

DD superfamily-mediated SMOC formation has been studied most intensively via structural studies of an inflammasome composed of Nod-like receptor (NLR), inflammatory caspase-1, and the ASC adaptor molecule^51–55^. Each NLRP family protein and caspase-1 contains a PYD and CARD at their respective N-terminus. ASC is a bipartite adaptor containing a PYD and CARD, which can link NLRP with caspase-1 by mediating the interaction of PYD–PYD through NLRP and the interaction of CARD–CARD through caspase-1. The results of an analysis based on cryo-EM indicated that all members of the DD superfamily in the inflammasome form a helical filament structure^19,21,48,56^.

**Fig. 2:**
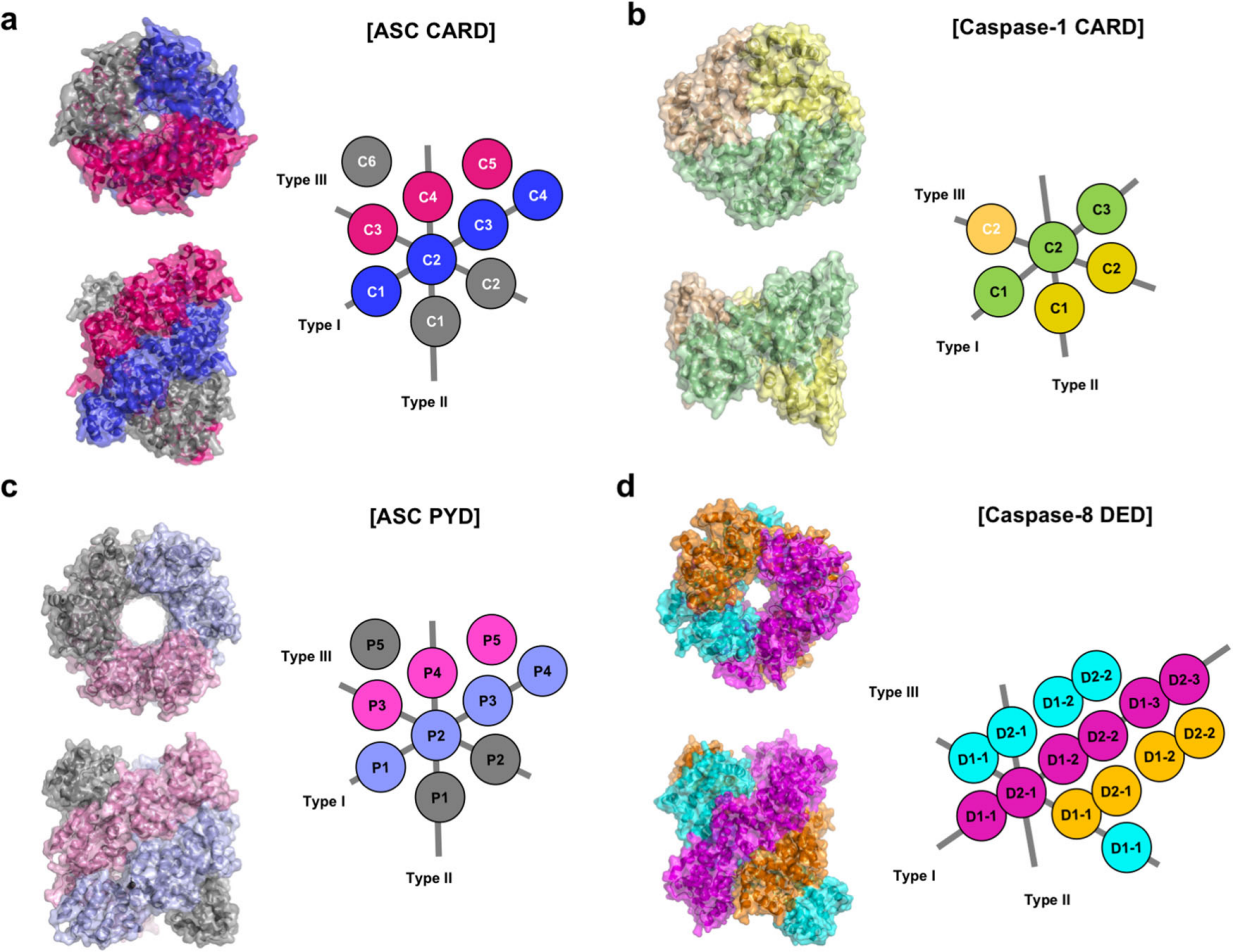
Helical filament structure of the death domain (DD) superfamily. Helical filament structures of **a** the ASC caspase recruitment domain (CARD), **b** caspase-1 CARD, **c** the ASC pyrin domain (PYD), and **d** the caspase-8 death effector domain (DED) are represented. The surface structures are shown in the left panel from a topside view. A schematic planar diagram is shown in the right panel. The three different types of interactions used for helical filament formation are designated type I, type II, and type III. C and P in the schematic model indicate CARD (C) and PYD (P), respectively. The tandem DEDs of caspase-8 that produce a helical filament structure are separately indicated by D1 for the first DED and D2 for the second DED in the schematic model.

The ASC CARD forms a helical filament with a diameter of ~8 nm and a central hole of ~1 nm (Fig. 2a)^19^. The filament architecture is assembled via left-handed one-start helical symmetry with approximately 3.6 subunits per turn. It is formed by three types of typical asymmetric interactions and displays threefold symmetry along the helical axis (Fig. 2a). Another CARD filament is introduced by caspase-1 to form a helical filament with a left-handed one-start helical assembly with approximately four subunits per turn. This structure is similar to the filament structure of ASC CARD (Fig. 2b)^21^. A caspase-1 CARD filament is also constructed using the three previously defined types of asymmetric interactions and has a diameter of 8 nm with an inner hole of approximately 1 nm, which is slightly larger than that of the ASC CARD^21^. ASC PYD also forms a helical filament using three types of canonical interaction modes. The filament is a right-handed helical filament with threefold symmetry along the helical axis (Fig. 2c)^48^. Although the diameter of the ASC PYD filament is similar to that of ASC CARD and caspase-1 CARD, the size of its inner hole was much greater (Fig. 2c). Based on the filament structures of the ASC CARD, caspase-1 CARD, ASC PYD, and NLRP PYD, the overall structure of the inflammasome and the assembly mechanism are proposed^48,57,58^. NLRP activation by a pathogen-associated molecular pattern triggers NLRP PYD assembly and filament formation, thus mediating ASC filament formation by serving as a nucleation platform via the PYD–PYD interaction. The helical filament of ASC recruits procaspase-1 via the CARD–CARD interaction. The recruited procaspase-1 also forms a helical filament in the inflammasome complex, which mediates proximity-induced self-activation^48,57,58^.

The only helical filament structure of DED was revealed via a structural study of the tandem DED in caspase-8, which is activated in the death-inducing signaling complex (DISC)^18^. The overall structure and construction strategies of the helical DED filament, formed by typical type I, II, and III interactions, are similar to those of other subfamilies. However, the DED filament diameter of ~9 nm is slightly greater than that of other filaments, and the right-handed helix does not have a distinct symmetry (Fig. 2d). In addition to these representative helical filament structures of the DD superfamily, various cases have been reported to date and are summarized in Table 1.

**Table 1:** Structures of various forms of DD superfamily.

DD superfamily	Proteins	Monomer	Dimer	Filament	Domain swapping
DD	FADD	O (2GF5)		(3OQ9)^a^	
	Fas	O (1DDF)		(3OQ9)^a^	O (3EZQ)
	RAIDD	O (2O71)		(2OF5)^a^	
	PIDD			(2OF5)^a^	
	TRADD	O (5XME)			
	RIP1	O (6AC5)			
	IRAK-2			(3MOP)^a^	
	IRAK-3	O (5UKE)			
	IRAK-4	O (2A9I)		(3MOP)^a^	
	Ankyrin-1	O (2YQF)			
	Ankyrin-2	O (4D8O)			
	Ankyrin-3	O (4O6X)			
	TNFRSF1A	O (1ICH))			
	TNFRSF16		O (2N83)		
	TNFRSF25	O (5YGS)			
	MALT1	O (2G7R)	O (6GK2)		
	MyD88			(3MOP)^a^	
	NF-kB-1	O (2DBF)			
	NF-kB-2	O (2D96)			
	THOC1	O (1WXP)			
	UNC5B	O (3G5B)	O (1WMG)		
DED	FADD	O (2GF5)			
	Caspase-8	O (5JQE)		O (5L08)	O (6AGW)
	PEA-15	O (6P6B)			
CARD	Caspase-1			O (5FNA)	
	Caspase-9		O (3YGS)		
	Apaf-1	O (2YGS)	O (3YGS)		
	ARC	O (4UZ0)			
	ASC	O (2KN6)		O (6N1H)	
	RAIDD	O (3CRD)			
	RIPK2	O (2N7Z)	O (2N83)	O (6GGS)	
	NLRP1	O (4IFP)			
	NLRC4	O (4KXF)		O (6N1I)	
	NOD1	O (2B1W)	O (2NSN)		O (2NSN)
	NOD4	O (2MJM)			
	CARMA1	O (4LWD)	O (4JUP)		
	ICEBERG	O (1DGN)			
	BinCARD	O (4DWN)			
	CARD8	O (4IKM)			
	CARD9	O (6E26)	O (6N2M)	O (6N2P)	O (6N2M)
	BIRC2	O (2L9M)			
	MAVS	O (2VGQ)		O (3J6J)	
	DDX58	O (2LWD)		O (4P4H)	
	Bcl-10	O (2MB9)	O (6GK2)	O (6BZE)	
PYD	ASC	O (2KN6)		O (3J63)	
	NLRP1	O (1PN5)			
	NLRP3	O (3QF2)			
	NLRP4	O (4EWI)			
	NLRP6	O (6NDJ)		O (6NCV)	
	NLRP7	O (2KM6)			
	NLRP10	O (2M5V)			
	NLRP12	O (2L6A)	O (4XHS)		
	NLRP14	O (4N1L)	O (4N1J)		O (4N1J)
	POP1	O (2HM2)			
	AIM2	O (4O7Q)		O (6MB2)	
	MEFV	O (2MPC)			
	MNDA	O (5WQ6)			

^a^Indicates the formation of helical oligomer rather than filaments.

### Domain-swapping-mediated dimerization of DD superfamily members

**Fig. 3:**
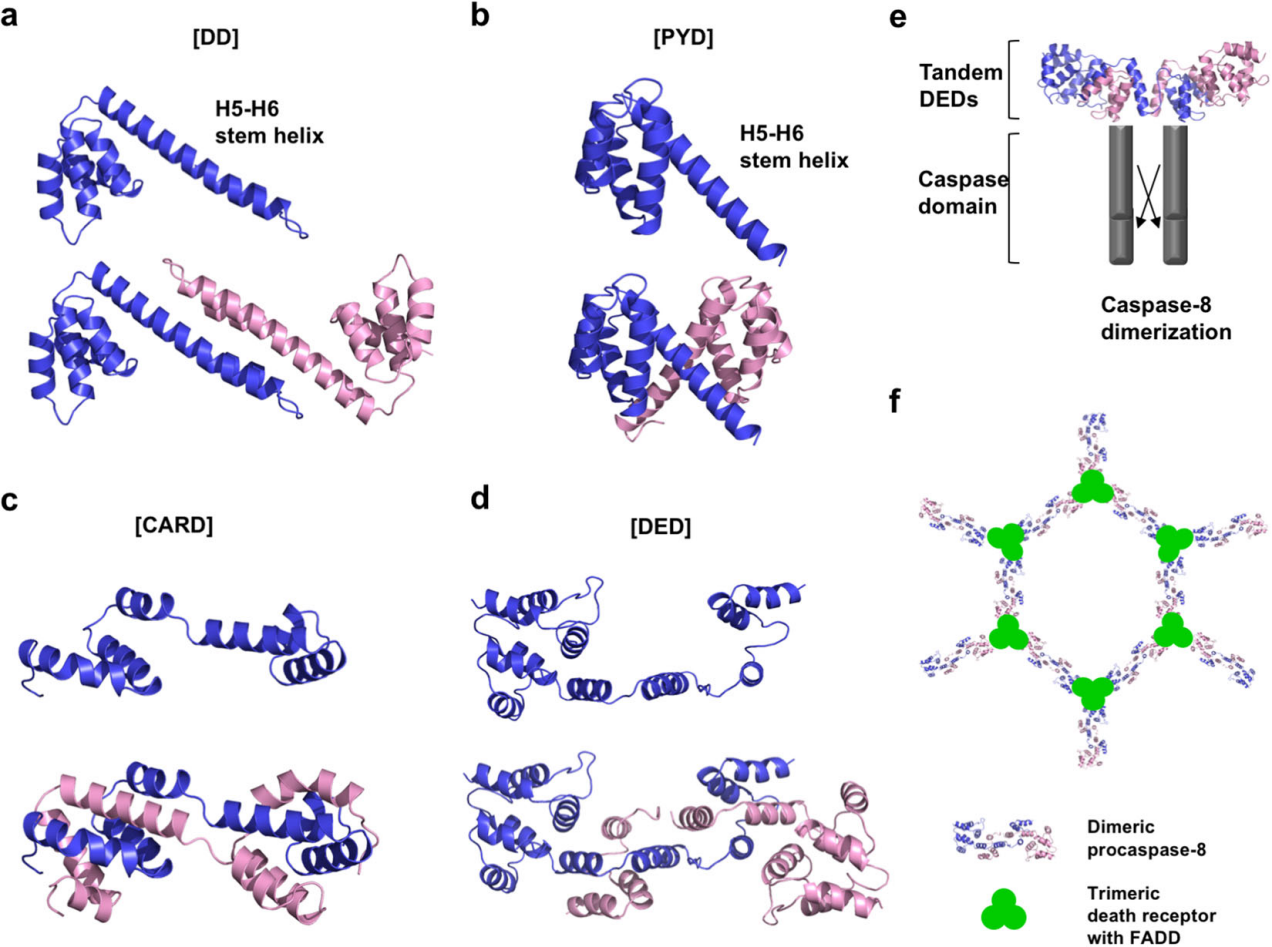
Domain-swapping-mediated oligomerization in death domain (DD) superfamily members. Representative cases of **a** DD (Fas DD), **b** pyrin domain (PYD) (NLRP14), **c** the caspase recruitment domain (CARD) (COP from frog virus 3), and **d** death effector domain (DED) (caspase-8) are shown. Stem helix-mediated dimerization was observed for a DD and **b** PYD. The connection of H5 and H6, which serves as a dimerization stem helix, is labeled. A monomer (upper panel) and a stem loop-mediated dimer (lower panel) are shown in a cartoon. Regional domain-swapping-mediated dimerization is shown for CARD (**c**) and DED (**d**). A monomer (upper panel) and regional domain-swapping-mediated dimer (lower panel) are shown using a cartoon. **e** Proposed activation model of caspase-8 by domain-swapping-mediated dimerization of the caspase-8 prodomain (tandem DEDs). **f** Tentative model of DISC chain assembly via the interaction of the trimeric death receptor bound to the death ligand, adaptor FADD, and domain-swapping-mediated dimerized caspase-8.

Domain swapping is widely used by proteins for the functional interconversion of monomers, dimers, and higher oligomers. It is also utilized by DD superfamily members for dimerization^59–64^. Domain-swapping-mediated dimerization has been observed in all subfamilies of the DD superfamily, including Fas DD^22^, NLRP14 PYD^65^, caspase-8 DED^66,67^, and vPOP CARD^68^. Based on structural studies, it was revealed that two different domain-swapping mechanisms can be used to form a stable DD superfamily dimer. The first mechanism involves stem helix (formed by connected H5 and H6)-mediated domain swapping, during which structural changes occur in the H5 and H6 regions. The stem helix is formed by H5 connecting to H6, which can interact with the stem helix through its binding counterpart. The DD and PYD subfamily members also use this mechanism for domain swapping. The details of this stem helix-mediated oligomerization were described in a structural study on the core of the DISC, which is composed of Fas DD/FADD DD (Fig. 3a)^22^. In this study, a structural alteration was observed in the H5 and H6 regions of the Fas DD, which formed a stem helix and mediated domain swapping. This was the critical step for Fas DD dimerization and for the binding of Fas DD to FADD DD to form the DISC core. This stem helix-mediated domain swapping that leads to Fas DD dimerization was suggested to be an important regulatory mechanism for DISC formation. Stem helix formation that leads to domain swapping was also found during a structural study of NLRP14 PYD^65^. Similar to the structural alteration of the Fas DD, NLRP14 PYD forms a combined H5−H6 stem helix, which mediates the dimerization of NLRP14 PYD (Fig. 3b). Although the interface of the stem helix during the dimerization of NLRP14 PYD is not related to that of the Fas DD, the structural change-mediated dimerization strategy is similar to that utilized by Fas DD in the DISC. This similarity is based on the stem helix formed by H5 and H6, which is critical for generating the dimer interface. NOD1 CARD is also dimerized using this mechanism^69^.

Another domain-swapping mechanism used by the DD superfamily is regional structural alteration-mediated local domain swapping, as demonstrated via structural studies of the DED and CARD subfamilies. In these cases, large structural changes involving the unfolding and extension of the H3−H6 region that allow for domain swapping were observed^70,71^. This dimerization strategy was shown in a structural study of viral CARD-only protein (vCOP)^68^. In this structure, H4, H5, and H6 in the typical six-helix vCOP bundle are displaced and inserted between H1, H2, and H3 in a molecule opposite of vCOP. This insertion forms the typical six-helix bundle structure, leading to vCOP dimerization (Fig. 3c). Another case of huge structural change-mediated domain swapping and dimerization in the DD superfamily was discovered during a structural study of tandem DEDs in caspase-8^66,67^. This study showed that the helical bundle of DED2 is unfolded from H4 to H6 and translocated to a counterpart molecule where it interacts with the H1, H2, and H3 helix bundles in the original H4−H6-contributing molecule, forming a new six-helix bundle (Fig. 3d). Both DED1 and DED2 in the caspase-8 tandem DEDs have an FL motif; it has been established that the FL motif in DED2 is critical for dimerization, oligomerization, and the further activation of caspase-8, whereas the FL motif in DED1 is not associated with either oligomerization or caspase activation^72–74^. This result explains the reason that the FL motif in DED2 is critical for caspase-8 activation, showing that it is the primary mediator for caspase-8 domain swapping and dimerization (Fig. 3e). By observing the domain-swapping-mediated dimerization of caspase-8, a model of DISC assembly and caspase-8 activation was suggested^66^. DISC assembly via the death ligand interaction with the death receptor, followed by FADD recruitment, mediates the accessibility of the FL motif in procaspase-8 DED2. This induced accessibility is followed by domain swapping, dimerization, and the activation of caspase-8. Because the trimeric death ligand and death receptor commonly interact, the complete DISC complex is thought to form a DISC cluster in lipid rafts (Fig. 3f).

## Structure and assembly strategies of the CIDE domain

Five CIDE domain-containing proteins that perform critical roles in apoptosis and energy metabolism (CIDE-A, CIDE-B, CIDE-3 (or CIDE-C and FSP27 in mice), DFF45 (ICAD in mice), and DFF40 (CAD in mice)) were identified and studied^9,11,12,14,75–77^. DFF40 and DFF45, primary executioners for apoptotic DNA fragmentation, contain a CIDE domain in their respective N-terminus^12,78^. The CIDE domain also forms a filament-like structure that is relevant to its function^24^. In Drosophila, the four CIDE domain-containing proteins DREP1–4 mutually control apoptotic DNA fragmentation^13^. Among these proteins, DREP4 is a homolog of human DFF40, an endonuclease that directly cuts chromosomal DNA during apoptosis^79,80^.

**Fig. 4:**
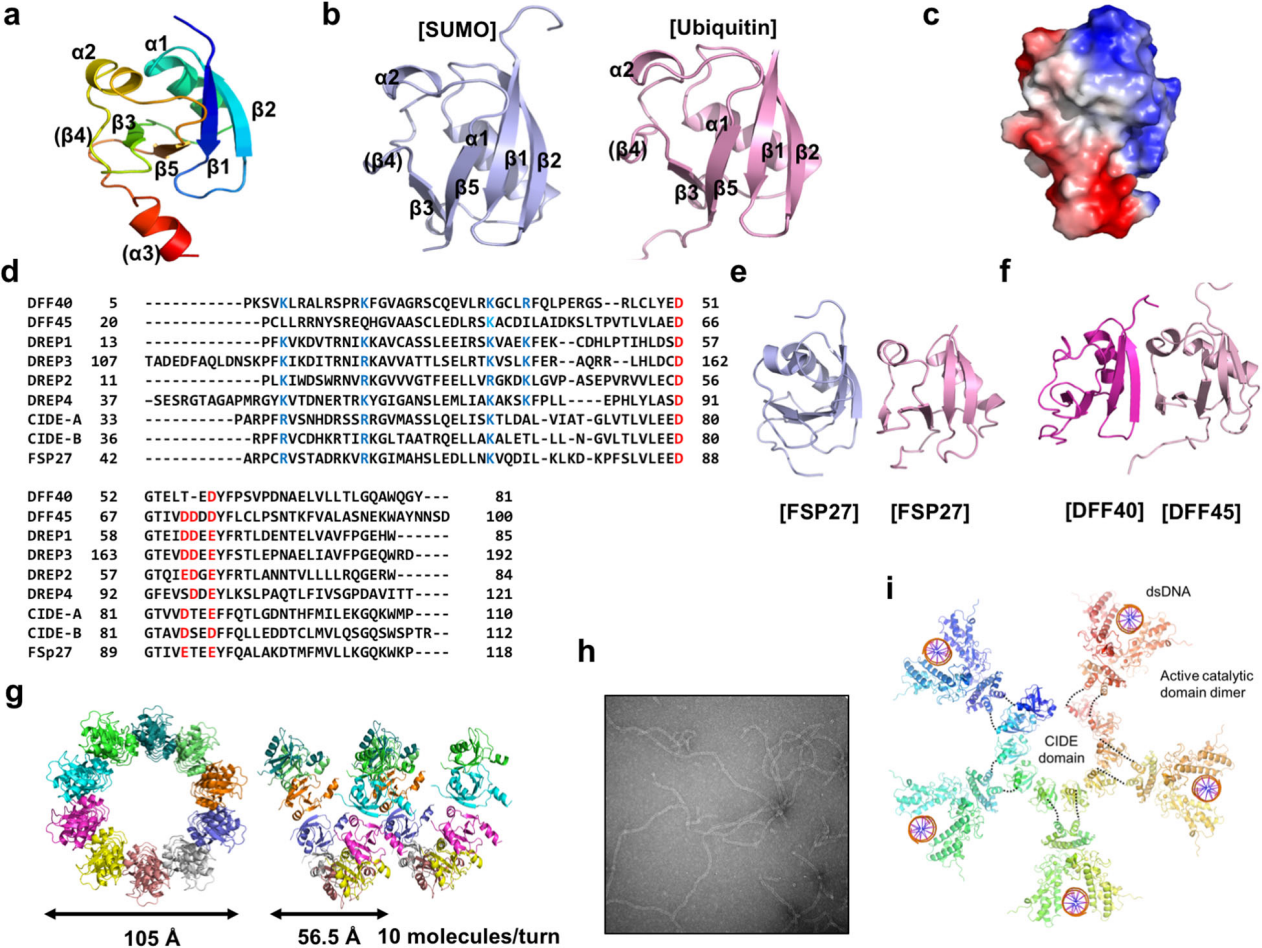
Various forms of the CIDE domain and their functional implications. **a** Typical structure of the CIDE domain. A representative DREP4 CIDE domain is presented. Secondary structures are indicated on the corresponding structure. Rainbow colors indicate the N-terminus in blue and the C-terminus in red. **b** Structural homologs of the CIDE domain. **c** The common surface feature of the CIDE domain, which contains distinct acid and basic patches on opposite sides. The DREP4 CIDE domain generates an electrostatic surface charge distribution. **d** Sequence alignment between various CIDE domains. Conserved charged residues involved in the formation of acidic and basic patches are indicated in red and blue, respectively. **e** The prototype homodimeric structure of the CIDE domain. The homodimeric CIDE structure is presented based on the structure of the FSP27 CIDE domain. **f** The prototype heterodimeric structure of the CIDE domain. The DFF40 CIDE and DFF45 CIDE complexes are representative of the heterodimeric interactions of CIDE domains. **g** Helical filament assembly of the CIDE domain was revealed by a structural study of the DREP4 CIDE domain. The top and side views of the DREP4 CIDE helical filament structure are shown in the left and right panels, respectively. **h** CIDE helical filament formation in solution. Negative stained images by electron microscopy show DREP4 CIDE filament formation in the solution. **i** Proposed model of DFF40 filament assembly during apoptotic DNA fragmentation.

The CIDE domain has an α/β-roll fold with two α-helices and five β-strands (Fig. 4a)^81–83^. This fold is similar to that of SUMO and ubiquitin, which are small protein modification proteins (Fig. 4b)^84,85^. The most distinct feature of the CIDE domain is its complementarity between the two opposing acidic and basic surfaces, which can support the formation of homodimeric and heterodimeric filament structures via head-to-tail polymerization (Fig. 4c)^86–89^. The results from a sequence analysis showed that the residues involved in basic and acidic surface formation are conserved in the CIDE domain, indicating that this type of charge distribution, creating two distinctly opposing surfaces, is common in this domain (Fig. 4d).

Representative CIDE domain homodimer formation was revealed via a structural study of FSP27 CIDE^87,90^. The interface was formed via residues on β1, β2, and α1 in one CIDE domain and residues on β4 and α2 in another binding CIDE domain (Fig. 4e). As expected, the homodimer interface was composed predominantly of electrostatic interactions formed by R46, R55, and K56 (basic patch) in one FSP27 molecule and E87, D88, and E93 (acidic patch) in the bound FSP27 molecule^87,90^. Although the functional relevance of FSP27 dimerization remains unclear, it may be critical to the biological activity of FSP27. The heterodimerization process of the CIDE domain is similar to that of the homodimerization process, as the CIDE domain has complementarity with the two opposing surfaces. According to the complex structures of the DFF40 CIDE and DFF45 CIDE domains, the basic patch (formed by K9, K18, K32, and R36 in the DFF40 CIDE domain) interacts with the acidic patch (formed by D66, D71, D72, and D74 in the DFF45 CIDE domain), indicating that this oppositely charged surface interaction is a common strategy for CIDE domain dimer formation (Fig. 4f)^78^.

The helical assembly mechanism of the CIDE domain has been recently revealed through structural study of the CIDE domains in DREP4 and DREP2^24,89^. This newly discovered helix is constructed with ten subunits with a 56.5 Å rise/turn and a diameter of 105 Å (Fig. 4g). This helical oligomer is formed by repetitive head-to-tail oligomerization of highly charged interfaces, detected, and then introduced to homodimeric and heterodimeric CIDE complexes. In the case of DREP4, a negatively charged patch formed by residues D91, E94, D97, E99, and D116 generates massive salt bridges and hydrogen bonds with a positively charged patch comprising residues K51, R59, K60, and K74 on the opposite molecule^24^. This helical structure formed by the CIDE domain was also formed in solution, as observed by EM (Fig. 4h). The helical assembly of the CIDE domain was also shown via structural studies of DREP2 and FSP27, indicating that this head-to-tail polymerization via charge−charge interactions may be an assembly mechanism common to CIDE domains^24,89^.

The function of this common head-to-tail helical CIDE domain assembly was studied by observing the apoptotic DNA fragmentation process executed by DFF40^24^. This functional study and previous biochemical studies showed that the CIDE domain-mediated DFF40 dimer has limited nuclease activity, possessing insufficient activity for fast chromosomal DNA fragmentation during apoptosis^9,91–93^. In contrast, the helical filament assembly of DFF40 via its N-terminal CIDE domain can increase the local concentration of DFF40 to impose a compatible distance, functioning as a molecular ruler to efficiently cleave the chromosomal DNA producing apoptotic DNA ladders (Fig. 4i)^24^. The various forms of the CIDE domain as revealed by structural studies are summarized in Table 2.

**Table 2:** Structures of various forms of CIDE domain.

CIDE proteins	Monomer	Dimer	Filament	PDB
CIDE-A	O			2EEL
CIDE-B	O			1D4B
FSP27		Homodimer	O	4MAC
DREP2			O	4D2K
DREP4			O	5XPC
CAD		Heterodimer		1F2R
ICAD		Heterodimer		1F2R
DFF40		Heterodimer		1IBX
DFF45		Heterodimer		1IBX

## Summary and outlook

The concept of modern signal transduction has been further advanced from classical signal transduction via the discovery of various SMOCs, which are location-specific, higher-order signaling complexes. This new signaling platform is especially important for cell death and innate immune signaling^94^. In particular, the DD superfamily, composed of the DD, CARD, PYD, and DED subfamilies, mediates SMOC formation using a common helical assembly mechanism. This SMOC can mediate signal transduction, signal amplification, and proximity-induced enzyme activation. DD superfamily-mediated SMOC formation includes caspase activating complexes such as an inflammasome for caspase-1 activation, a PIDDosome for caspase-2 activation, an apoptosome for caspase-9 activation, and a DISC for caspase-8 and caspase-10 activation. A SMOC also includes various signaling platforms, such as a MYDosome for Toll-like signaling and CMD complexes for immune cell signaling. Understanding these huge molecular complexes can explain proximity-mediated allosteric enzyme activation, cooperativity, signal amplification, threshold behavior, and the spatial and temporal control of signal activation and termination.

In addition to helical filament assembly by DD superfamily members, various assembly mechanisms, including several different types of dimerization, have also been observed. Among these formation strategies, domain-swapping-mediated dimerization is a newly identified alternative for DD superfamily dimerization. As DD superfamily-containing proteins are functionally diverse, acting as enzymes or scaffolding adaptors in various cellular signaling pathways, it is unsurprising that these proteins have evolved the capability to form many different types of oligomers using various oligomerization strategies^18,24,47,48,95,96^. Recent studies have demonstrated that CIDE domain-containing proteins also form higher-order SMOCs that perform apoptotic DNA fragmentation functions^24^.

Domain-mediated protein interactions, especially SMOC-mediated signaling transfer in cell death and innate immunity, are important for understanding signal transduction. Signal transduction failure is linked to various human diseases, such as gout, multiple sclerosis, neurodegenerative diseases, diabetes, and Crohn’s disease. Understanding the emerging concept of signal transduction by the formation of the large molecular scaffolding center SMOC via DD superfamily members and the CIDE domain may provide a new target for treating human diseases. Blocking SMOC assembly, therefore, may be an interesting avenue for further research.
